# Effects of isoflurane anesthesia on resting‐state fMRI signals and functional connectivity within primary somatosensory cortex of monkeys

**DOI:** 10.1002/brb3.591

**Published:** 2016-10-11

**Authors:** Tung‐Lin Wu, Arabinda Mishra, Feng Wang, Pai‐Feng Yang, John C. Gore, Li Min Chen

**Affiliations:** ^1^ Vanderbilt University Institute of Imaging ScienceNashvilleTNUSA; ^2^ Biomedical EngineeringVanderbilt UniversityNashvilleTNUSA; ^3^ Radiology and Radiological SciencesVanderbilt UniversityNashvilleTNUSA

**Keywords:** fMRI, hand, non‐human primates, resting state, somatosensory system

## Abstract

**Introduction:**

Correlated low‐frequency fluctuations of resting‐state functional magnetic resonance imaging (rsfMRI) signals have been widely used for inferring intrinsic brain functional connectivity (FC). In animal studies, accurate estimate of anesthetic effects on rsfMRI signals is demanded for reliable interpretations of FC changes. We have previously shown that inter‐regional FC can reliably delineate local millimeter‐scale circuits within digit representations of primary somatosensory cortex (S1) subregions (areas 3a, 3b, and 1) in monkeys under isoflurane anesthesia. The goals of this study are to determine (1) the general effects of isoflurane on rsfMRI signals in the S1 circuit and (2) whether the effects are functional‐ and regional‐ dependent, by quantifying the relationships between isoflurane levels, power and inter‐regional correlation coefficients in digit and face regions of distinct S1 subregions.

**Methods:**

Functional MRI data were collected from male adult squirrel monkeys at three different isoflurane levels (1.25%, 0.875%, and 0.5%). All scans were acquired on a 9.4T magnet with a 3‐cm‐diameter surface transmit‐receive coil centered over the S1 cortex. Power and seed‐based inter‐regional functional connectivity analyses were subsequently performed.

**Results:**

As anesthesia level increased, we observed (1) diminishing amplitudes of signal fluctuations, (2) reduced power of fluctuations in the low‐frequency band used for connectivity measurements, (3) decreased inter‐voxel connectivity around seed regions, and (4) weakened inter‐regional FC across all pairs of regions of interest (digit‐to‐digit). The low‐frequency power measures derived from rsfMRI signals from control muscle regions, however, did not exhibit any isoflurane level‐related changes. Within the isoflurane dosage range we tested, the inter‐regional functional connectivity differences were still detectable, and the effects of isoflurane did not differ across region‐of‐interest (ROI) pairs.

**Conclusion:**

Our data demonstrate that isoflurane induced similar dose‐dependent suppressive effects on the power of rsfMRI signals and local fine‐scale FC across functionally related but distinct S1 subregions.

## 1 Introduction

Since the discovery of temporally correlated low‐frequency fluctuations of magnetic resonance imaging (MRI) signals in a resting state, rsfMRI has been widely used for assessing functional connectivity between brain regions, identifying functional circuits, and probing alterations in brain functional networks under various conditions in both humans and animals (Biswal, Yetkin, Haughton, & Hyde, 1995; Chen et al., 2011; Fox, Corbetta, Snyder, Vincent, & Raichle, 2006; Fox & Greicius, 2010; Vincent et al., 2007; Wang et al., 2013). Identification of similar functional circuits in awake humans and anesthetized monkeys has led to a wide recognition that resting‐state functional connectivity (rsFC) is a fundamental feature of the brain's functional organization, while recent reports of similar correlations in the spinal cord (Chen, Mishra, Yang, Wang, & Gore, 2015) suggest that they extend to other parts of the nervous system. The use of anesthesia is necessary in many animal fMRI studies to reduce motion‐related artifacts and distress. Some human studies have also used anesthesia to understand its general effects on brain activity and consciousness, particularly in pediatric populations. Understanding the effects of anesthesia on rsFC could improve our understanding of the relationship between BOLD (blood oxygenation level dependent) signal changes and underlying neural activity, and assist our interpretation of rsfMRI signals in anesthetized subjects.

Anesthesia is known to suppress neural activity in the brain (see a review paper, Heinke & Koelsch, 2005) as well as affect fMRI signals in both task‐engaged and resting states. Numerous studies have reported primarily suppressive effects of anesthesia on both, neural electrophysiological and fMRI signals, which are agent‐ (anesthetic), dose‐, and circuit‐specific. (Grandjean, Schroeter, Batata, & Rudin, 2014; Jonckers et al., 2014). The general notion is that higher‐order brain regions involved in cognitive functions are more sensitive to the influence of anesthesia, whereas lower‐order sensory regions are less affected. For example, under conscious sedation with midazolam, functional connectivity of the posterior cingulate cortex (PCC) within the global default‐mode network (DMN) in humans was significantly reduced (Greicius et al., 2008). Similarly, Liu, Zhu, Zhang, & Chen (2013) reported that resting‐state networks in rat brains remain measurable under light isoflurane anesthesia, but become less spatially specific at deeper anesthesia levels. Most of these studies have examined the effects of anesthesia on large global scale networks (in centimeter(s) size). However, little is known about the effects of anesthesia on local meso‐scale circuits (in millimeters) FC, and whether these effects are functionally and regionally specific.

Our previous rsfMRI studies of the brain and spinal cord in non‐human primates (squirrel monkeys) demonstrated that under light‐to‐moderate (around 1.0%) anesthesia with isoflurane, neuronal spiking, local field potential, and rsfMRI functional connectivity measures were robust and sensitive in differentiating functionally distinct cortical regions and local fine‐scale sensory circuits within the digit and face representations of primary (areas 3a, 3b, 1, and 2) and secondary (S2) somatosensory cortices as well as gray matter horns of the spinal cord (Chen et al., 2015; Wang et al., 2013; Wilson, Yang, Gore, & Chen, 2016; Yang, Qi, Kaas, & Chen, 2014). Importantly, within these circuits, we identified close relationships between strength of inter‐regional rsFC, synchrony of baseline spontaneous spiking and local field potential activity (Wang et al., 2013; Wilson et al., 2016). This observation indicates that changes in rsfMRI signals indeed correlate with changes in neuronal activity within the S1 circuit. Furthermore, under the same isoflurane anesthesia condition, we also showed that rsfMRI signals within gray matter of cervical spinal cord were able to reveal traumatic injury‐induced rsFC changes (Chen et al., 2015). Few studies have explored into graded effects of anesthesia (Barttfeld et al., 2014; Hutchison, Hutchison, Manning, Menon, & Everling, 2014; Liu, Zhu, et al., 2013; Liu, Pillay, et al., 2013) and even so, none has investigated inter‐regional functional connectivity within a local functionally specific cortical network. To date, from the functional circuit perspective, there are at least three critical questions remaining that concern the use of rsfMRI for indicating functional connectivity under anesthesia: (1) Is the anesthesia effect universal or regional‐ and circuit‐specific? (2) Does anesthesia have different effects on functionally related but distinct cortical regions? and (3) in what anesthesia range is rsFC still a sensitive and robustly reliable measure? To address these questions, we quantified the effects of anesthesia with two metrics: power analysis (calculating low‐frequency fluctuation power (ALFF) and fractional power (fALFF) (Zou, Wu, Stein, Zang, & Yang, 2009)) of the rsfMRI signal in the entire S1 region without any assumptions, and seed region‐based functional connectivity analyses (see a review paper, Lee, Smyser, & Shimony, 2013), with a presumption that cortical regions involved together in the processing of external inputs also exhibit strong functional connectivity at rest. Overall, our study provides novel insights into graded anesthesia effects on functionally closely related meso‐scale local circuits.

## 2 Materials and Method

### 2.1 Animals and preparation

Two male adult squirrel monkeys were studied. Each animal was sedated with ketamine hydrochloride (10 mg/kg)/atropine (0.05 mg/kg) and was then maintained with isoflurane anesthesia (0.5–1.25%) delivered in a 70:30 N_2_O/O_2_ mixture. After intubation, each animal was placed in a custom‐designed MR cradle where the head was secured with ear and head bars to prevent any motions. The animal was mechanically ventilated (40 bpm), monitored, and infused intravenously with 2.5% dextrose in saline solution (3 ml kg^−1^ hr^−1^) throughout the entire imaging session. Vital signs of the animal including peripheral oxygen saturation and heart rate, EKG, end‐tidal CO_2_, and respiratory pattern were continuously monitored and recorded. Temperature of the animal was also kept between 37.5 and 38.5°C with a circulating water blanket. All procedures were in compliance with the Society for Neuroscience guidelines for animal use in research and were approved by the Institutional Animal Care and Use Committee (IACUC) at Vanderbilt University.

### 2.2 Variation in isoflurane levels

We altered the levels of isoflurane (from high to low) and functional MRI data were collected after the animal's physiological condition was stable. At least three sets of functional MRI scans at each isoflurane level (1.25%, 0.875%, and 0.5%) were acquired within each imaging session. After each change of isoflurane level, we allocated at least 10 min to allow the physiological conditions to stabilize before the next image acquisition. Stabilization of the anesthesia and animal's physiological condition was indicated by constant vital signals such as heart rate, end‐tidal CO_2_, and respiration patterns.

### 2.3 MRI data acquisitions

All scans were acquired on a 9.4T 21‐cm‐bore magnet (Varian Inc.) using a 3‐cm‐diameter surface transmit‐receive coil centered over the S1 cortex. Specifically, four 2‐mm‐thick oblique image slices were centered over the central and lateral sulci of either left or right hemisphere. Only the top slice, where S1 cortex resides, was analyzed in this study. High‐resolution structural images were also acquired using a gradient echo sequence (TR/TE = 200/16 ms, NEX = 6, flip angle = 30 degrees, resolution of 0.068 × 0.068 × 2 mm^3^). BOLD fMRI images were acquired using a T_2_*‐weighted GE‐EPI sequence (TR/TE = 1500/19 ms, 1 shot, resolution of 0.547 × 0.547 × 2 mm^3^, 1.5 s/volume) during both tactile stimulation and in a resting state (without stimulation). For each imaging session, multiple runs of the functional data were acquired in an interleaved manner at each different isoflurane levels. Each imaging run contained 300 image volumes. A vibrotactile stimulation of 8 Hz (0.34 mm vertical indentations of a probe 2‐mm‐diameter) of individual distal finger pads, as alternating 30 seconds off/on blocks, was delivered to elicit cortical activations and to allow identification of digit regions in S1 subregions of areas 3a, 3b, and 1.

### 2.4 fMRI data preprocessing

Slice timing corrections were performed with *spm8 *(SPM, RRID:SCR_007037) in Matlab (MATLAB, RRID:SCR_001622) followed by 2D slice‐by‐slice motion correction and spatial smoothing with a full width at half maximum of 0.8 mm. Motion correction parameters (two translations and one rotation) were considered as nuisance parameters to be regressed out of the time course data using a general linear model. Muscle voxels did not exhibit a trend in power across different isoflurane levels, so fMRI signals extracted from selected muscle voxels were subsequently also used as nuisance parameters to be regressed out for inter‐regional correlation analysis in order to minimize global physiology‐related signal variations. A temporal high‐pass filter (Type 2 Chebyshev IIR filter, cut‐off frequency 0.008 Hz) along with linear detrending was applied before resting‐state power analyses were performed. An additional low‐pass filter (Type 2 Chebyshev IIR filter, cut‐off frequency 0.08 Hz) was applied to the temporal signals for the inter‐regional resting‐state functional connectivity analysis.

### 2.5 Power analysis of resting‐state fMRI signals

For each run obtained from the two monkeys (*n *= 6, 7, 7 runs for 1.25%, 0.875%, and 0.5% isoflurane levels, respectively), ALFF (amplitude of low‐frequency fluctuations) and fALFF (fractional amplitude of low‐frequency fluctuations) analyses were performed. Specifically, power spectra of resting‐state fMRI signals of every voxel within the S1 region were estimated by transforming each fMRI signal time series into the frequency domain via the Fast Fourier Transform. Non‐brain voxels were masked out in the analysis. Because power is proportional to the square of the amplitude, the square root of the power spectra at each frequency was taken. The ALFF was then computed by summing amplitudes in the low‐frequency range (0.01–0.08 Hz). Likewise, fractional ALFF was calculated by expressing the ALFF as a ratio over the sum of amplitudes in the entire frequency range (0.01–0.33 Hz). fALFF and ALFF measures were subsequently normalized within each monkey by computing relative z‐scores before combining the runs for group analyses. Finally, boxplots for power analyses were generated using BoxPlotR (Spitzer, Wildenhain, Rappsilber, & Tyers, 2014).

### 2.6 Seed‐based inter‐regional functional connectivity analysis

Identification of seed regions is a critical step for inter‐regional correlation analysis. Seed at the single‐voxel level was defined as the one exhibiting the strongest response (highest t value) to single digit stimulation in each area. Stimulus evoked activation maps were generated in *spm8* with custom scripts (Chen et al., 2007). Briefly, the stimulus presentation paradigm was first convolved with a hemodynamic response function (HRF) using the HRF function in *spm8*. The output of the convolution was then used as the predictor (or model) in a subsequent voxel‐wise correlation analysis of fMRI BOLD time courses of the entire image volume. The strongest stimulus–responsive voxels (*p *< .001) were identified as seeds in areas 3b, 3a, and 1 for subsequent resting‐state analyses (Figure 4). One voxel in the neighboring area, 3b face location, was also identified as a control. The face region was determined by the overall somatotopic map of this hand‐face region, and the presence of neurons with face receptive fields were determined with electrophysiology. The functional responses of fMRI seeds were later confirmed with electrophysiology recordings (Figure 4) and those voxels were used for inter‐regional correlation analyses. Electrophysiology recordings were manually registered to structural images by overlaying locations of specific regions such as the lateral and central sulcus, and vessel landmarks. Pairwise correlation analysis was then performed on BOLD time courses extracted from different region‐of‐interest (ROI) seeds at different anesthesia levels. Correlation coefficients (*r* values) were calculated to indicate the strength of functional connectivity between two ROIs.

## 3 Results

### 3.1 BOLD signal time series at different isoflurane levels

**Figure 1:**
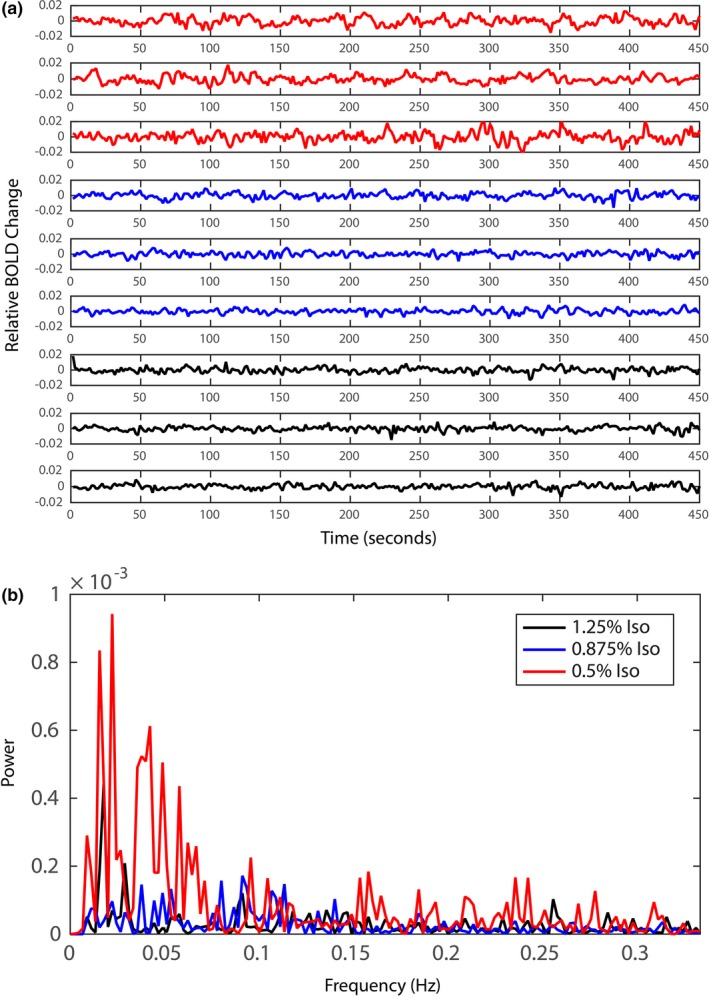
Blood oxygenation level‐dependent (BOLD) resting‐state signal time series and power spectral density at three different anesthesia levels. (a) Time series of a voxel in the somatosensory region in a representative animal of the same session. The monkey was under three different isoflurane levels: 1.25%, 0.875%, and 0.5% plotted in black, blue, and red lines, respectively, with three runs at each level. (b) Power spectral density estimates with the Fast Fourier Transform for one of the time series at 1.25%, 0.875%, and 0.5% isoflurane levels

Figure 1a displays three time series (from three imaging runs) of a selected voxel in the S1 region at three different anesthesia levels in one representative monkey. The consistently decreasing amplitudes of spontaneous signal fluctuations from low to high isoflurane levels (red: 0.5%, blue: 0.875%, black: 1.25%) are visible throughout the entire BOLD time series. Power spectral densities of the signal time series within 0–0.3 Hz frequency range at each isoflurane level for one representative run are shown in Figure 1b. The total power in the time series at 0.5% (red line) anesthesia is much greater than the ones with higher anesthesia of 0.875% (blue line) and 1.25% (black line). In addition, majority of the power is concentrated in the frequency range between 0.01 and 0.08 Hz, the low‐frequency range of resting‐state BOLD signals that is conventionally used in functional connectivity analyses. Overall, fMRI BOLD signals acquired at lower anesthesia levels present clearer hemodynamic signal fluctuations in the time series and larger power spectral densities in the lower frequency range.

### 3.2 Power analysis of resting‐state fMRI signals in the entire somatosensory S1 region

**Figure 2:**
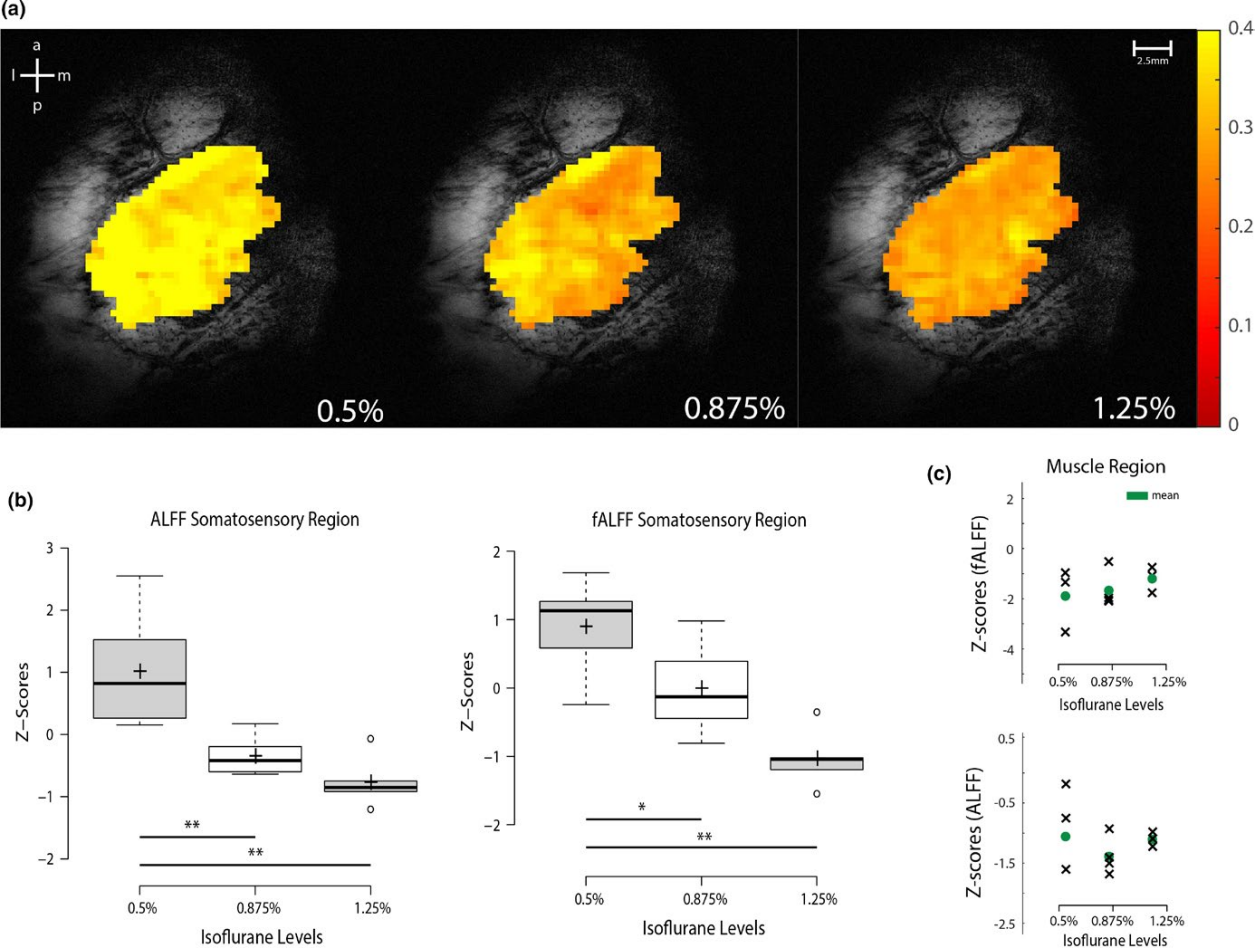
Relationship between the power of resting‐state fMRI blood oxygenation level‐dependent (BOLD) signals and level of anesthesia. (a) Representative fALFF maps of the somatosensory region under three different isoflurane levels. Color bar: range of fALFF values. (b) Group boxplots of the mean fALFF and ALFF in the somatosensory regions from two squirrel monkeys. Center lines of the boxplot represent the medians while the box limits present the 25th and 75th percentiles. Outliers are represented by the dots. Crosses indicate the means for 1.25% (*n *= 7 runs), 0.875% (*n *= 7), and 0.5% (*n *= 6) isoflurane levels, respectively. **p ≤ .005; *p ≤ .05 (nonparametric Mann–Whitney test) (c) Scatter plots of fALFF and ALFF measurements in the muscle control region in one monkey. Green dots indicate the mean values. a, anterior; l, lateral; m, medial; p, posterior

**Figure 3:**
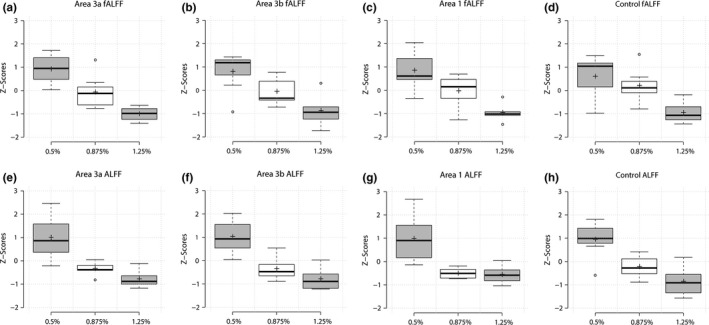
Effects of anesthesia level on fALFF and ALFF of different S1 subregions (areas 3a, 3b, and 1). Boxcar plots of group z‐scores of the fALFF (a–d) and ALFF (e–h) as a function of anesthesia level at specific regions of interest: area 1, area 3b, and area 3a digit regions and the area 3b face control region. Center lines of the boxplot represent the medians while the box limits present the 25th and 75th percentiles. Outliers are represented by the dots. Crosses indicate the mean for 1.25% (*n *= 7 runs), 0.875% (*n *= 7), and 0.5% (*n *= 6) isoflurane levels, respectively

Both fALFF and ALFF of the somatosensory region were calculated from the power spectral densities. fALFF maps from one representative monkey at different isoflurane levels are displayed in Figure 2a. Under an isoflurane level of 0.5%, almost the entire somatosensory region has a fALFF above 0.3 (see the color scale bar in Figure 2a). As the anesthesia level increased, the strength and number of pixels above 0.3 fALFF decreased (Figure 2a). Quantifications of the group fALFF and ALFF values (across runs and animals) in the S1 cortex revealed the same trend, that is, the magnitudes of low‐frequency signal fluctuations decreased monotonically as anesthesia level increased (Figure 2b). In contrast, control muscle regions showed no trends (Figure 2c) and overall weaker fALFF and ALFF values. The same power analyses were also performed on specific digit regions in area 3a, area 3b, area 1, and one face control region in area 3b at the single‐voxel level. The group results are presented in Figure 3. The same monotonically decreasing trend can be seen in all specific regions as a function of increasing anesthesia level, indicating a global increase in low‐frequency (0.01–0.08 Hz) power in the S1 region regardless of the functional specificity (e.g., hand vs. face regions) as anesthesia level was lowered.

### 3.3 Effects of anesthesia level on functional connectivity between region of interests

**Figure 4:**
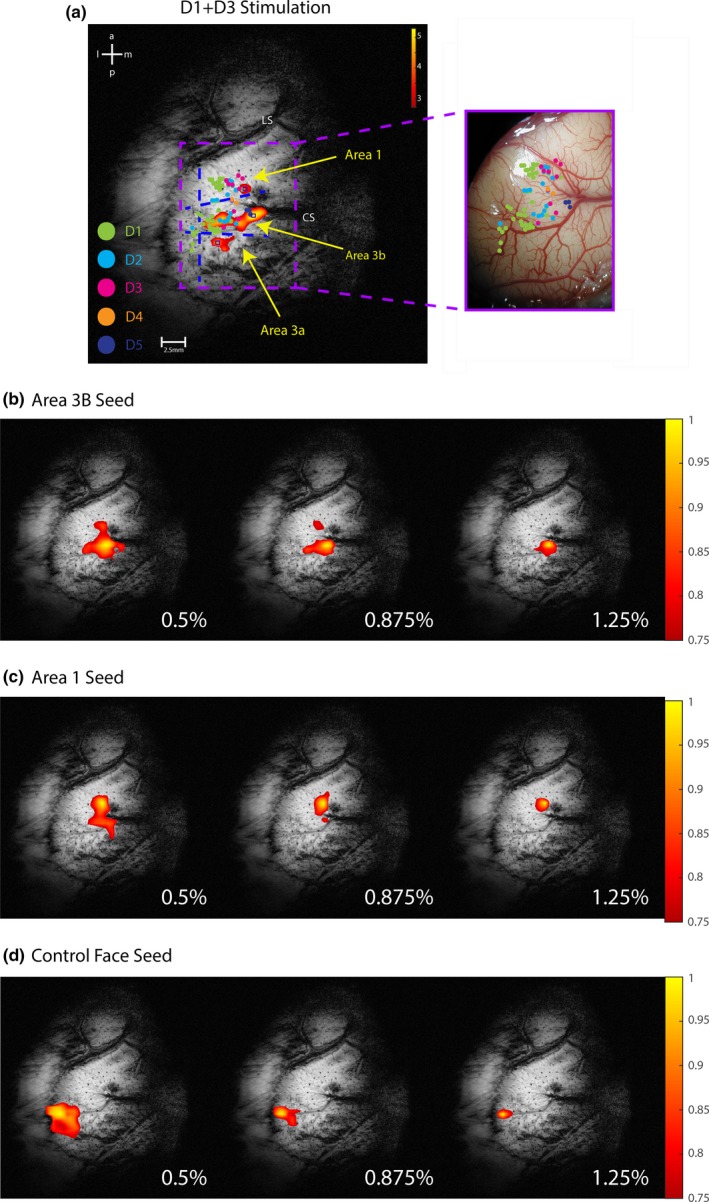
Tactile stimuli evoked fMRI activation map and region of interest (ROI) seed‐based voxel‐wise correlation maps within S1 cortex. (a) Left: blood oxygenation level‐dependent (BOLD) activation map (thresholded at *t *> 2.7) to simultaneous 8 Hz vibrotactile stimulation of digits 1 & 3. Scale bar: range of t values. Color dots represent the overlaid microelectrode penetration sites with color indicating the receptive field of neuron on individual digits. Horizontal dotted blue lines: estimated inter‐area borders. Vertical dotted blue line: estimated hand‐face border. CS and LS: central and lateral sulci. Voxels with the strongest response (highest *t* values) were chosen in areas 3b and 1 as seeds for rsFC analysis indicated by squared blue boxes. Right: aligned original electrophysiology map (brain surface blood vessel map). (b–d) Effects of isoflurane levels on the voxel‐wise correlation maps (thresholded at *r *> .75) of three ROI seeds (area 3b: b, area 1: c, face control: d) in one representative monkey. The yellow voxels indicate the locations of seeds. Scale bars indicate the range of r values

We further examined the effects of anesthesia level on inter‐regional correlation strengths, the measure that is most often used in resting‐state functional connectivity analyses. To ensure the functional specificity of each seed, we used the stimulus evoked fMRI activations as a reference (Figure 4a) for the selection of seeds. Tactile stimulation of D1 and D3 elicited strong fMRI signal changes in areas 3a, 3b, and 1. The locations of these activation foci were confirmed by microelectrode mapping and recording (right panel in Figure 4a). It is clear that D1 and D3 fMRI activation foci are located appropriately in the digit representation region defined by neuronal receptive field properties (see color‐coded dots in Figure 4a). At low 0.5% isoflurane level, a seed in area 3b showed high correlations with its immediate voxels within area 3b and area 1 (left image in Figure 4b). When the isoflurane level was increased to 0.875% and 1.25%, the number of highly correlated voxels decreased. The same phenomenon was observed in all three ROI seeds (Figure 4b–d) in both monkeys across multiple runs.

**Figure 5:**
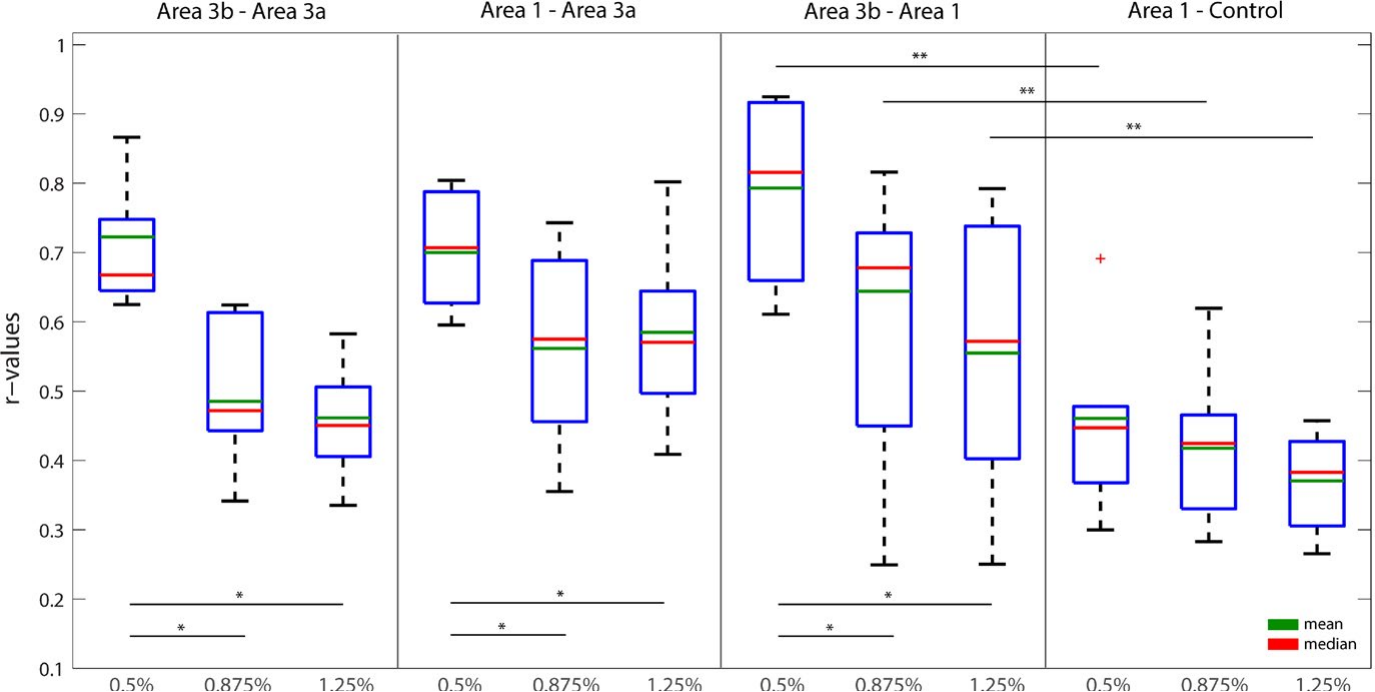
Group quantification of anesthesia effects on inter‐regional functional connectivity within S1 cortex. Boxplots of correlation strengths (*r* values) of four region‐of‐interest (ROI)‐pairs (from left to right: area 3b digit and area 3a digit; area 1 digit and area 3a digit; area 3b digit and area 1 digit; area 1 digit and face control) at three isoflurane levels. **p ≤ .005; *p ≤ .05 (nonparametric Mann–Whitney test). The red cross represents an outlier data point. Regardless of ROI pairs, anesthesia effects (at three different levels) were significant *p *<* *.0001 (two‐way ANOVA test)

Moreover, group level pairwise quantifications of the inter‐regional functional connectivity strengths (r values) between digit seeds in area 3b, area 1, and area 3a support the observation in the single subject (Figure 5). Importantly, the patterns of inter‐ROI pair variations in the functional connectivity strength are retained for all ROI pairs between digits and digit‐face control pairs (e.g., area 3 digit – area 1 digit vs. area 1 digit – face control) (right panel in Figure 5). Statistically, the dose‐dependent effect on functional connectivity between subregions of S1 cortex was analyzed using a one‐way analysis of variance (ANOVA). The one‐way ANOVA suggests that the levels of anesthesia have different effects on the response (functional connectivity measures, *p *< .0001). A nonparametric test (Mann–Whitney test Wilcoxon, MWW) was also implemented to evaluate the pairwise statistical significance of anesthesia effects on fALFF/ALFF and inter‐regional functional connectivity measures (*p *< .05 or *p *< .005) in addition to the standard multiple comparisons performed using ANOVA. Given the smaller size of the data, the MWW test is preferred because it does not assume a normal distribution of the input data, unlike the t‐test. A two‐way ANOVA was further implemented to evaluate the effects of two factors: i. levels of anesthesia and ii. types of inter‐ROI connection on the response variable. The test results suggest that the effects of anesthesia level on the three ROI pairs (area 1–3b, area 1–3a, area 3b–3a) are statistically significant (*p *< .0001). The effect of the second predictor (i.e., types of connection) was found to be insignificant, suggesting various inter‐ROI pairs do not explain the difference in functional connectivity. However, when the two‐way ANOVA was performed considering the digit‐face (control) resting‐state correlations as the second predictor, the *p* value was <.05. This clearly suggests that the effects of anesthesia measured in terms of resting‐state correlations between subregions of the S1 cortex are different from what we observed between the subregions and face area.

## 4 Discussion

In this study, we identified and computed how inter‐regional functional connectivity within a small cortical network (subregions within the primary somatosensory cortex, S1) varies as three different isoflurane levels were altered in a systematic manner. Our power and inter‐regional functional connectivity analyses provided a quantitative evaluation of anesthesia‐dependent functional connectivity in the S1 region. Our results also support the neuronal basis of rsfMRI signals, and the assessment of a suitable isoflurane range will be helpful for avoiding alterations of intrinsic functional relations between different regions in a resting state.

### 4.1 Graded effects of anesthesia on rsfMRI signals in fine‐scale circuit within S1 cortex

Previous studies aimed at understanding the effects of anesthesia have focused primarily on large‐scale functional connectivity networks, such as DMN or frontal–parietal networks (Greicius et al., 2008). Few studies have examined the effects of anesthesia on local fine‐scale functional circuits and addressed whether the degree of anesthesia influence varies across cortical regions with different levels of functional similarity. For example, Liu, Hirano, et al. (2013) examined before versus after anesthesia effects on primary (S1) and secondary (S2) cortex of marmosets. Another study also investigated anesthesia effects in forelimb and barrel field regions in the S1 cortex of rats (Liu, Zhu, et al., 2013). However, none has explored deep into specific fine subregions of the S1 cortex, and most studies have examined anesthesia effects in broad terms (before vs. after or light vs. deep), overlooking anesthesia's fine‐graded effects. Some investigations have begun to look into the dose‐dependent effects of anesthesia (Hutchison et al., 2014; Liu, Zhu, et al., 2013; Liu, Pillay, et al., 2013), but not specifically at a local fine‐scale circuit. We believe this information is important because it provides novel insights into anesthesia‐dependent effects on functionally similar regions and sizes of brain circuits. In this context, this study focused on a local circuit of subregions of S1 cortex (identified with stimulus‐driven activations and electrophysiology) and quantified the anesthesia effects at three different levels on four sets of ROIs: (1) entire S1 regions regardless of the functional specificity of its compartment, (2) functionally highly correlated hand regions, (3) functionally unrelated hand and face regions, and (4) nonneural muscle regions. Each ROI set was selected to represent a different level of functional similarity.

By measuring the power (ALFF and fALFF) of rsfMRI signals, we observed universal dose‐dependent suppressive effects of isoflurane anesthesia on cortical regions regardless of their functional heterogeneity or functional similarity. The amplitude of low‐frequency fluctuations (ALFF) (Zang et al., 2007) is a useful metric that computes total power within the low‐frequency range of 0.01–0.08 Hz, and has been widely used as a measure of resting‐state activity, providing spatial distributions of low‐frequency power. Fractional ALFF (fALFF) is the computed ALFF as a fraction of the entire frequency range (Zou et al., 2009). The fALFF has been reported to be less prone to artifacts such as pulsatile effects, while ALFF has also been shown to be more sensitive in picking out differences between individuals and group (Zuo et al., 2010). Global decreases in fALFF and ALFF were observed throughout the somatosensory region, including the specific ROIs of hand representation in areas 3a, 3b, and 1, and face region in area 3b, as anesthesia levels were increased (Figures 2 and 3). This indicates that increasing anesthesia diminishes spontaneous BOLD fluctuations throughout the S1 region, rather than shutting down specific neural circuits. A recent observational study in rhesus monkeys on dose‐dependent effects of anesthesia on regional activity have also demonstrated reduced ALFF in numerous cortical regions as anesthesia levels were raised (Lv et al., 2016). Consistent with the finding, we found that variation in anesthesia within a small physiological range affects the power and connectivity strengths of rsfMRI signals in S1 subregions. In contrast, such effects were not detected in muscle regions. The non‐dose‐dependent effects on muscle regions also indicate that isoflurane introduced universal (not functionally related) suppressive effects. Importantly, this differential effect of anesthesia on cortex and muscle indicate that the effects of isoflurane are universal regardless of brain regions, and so are very likely driven by changes in underlying neuronal activity. Similarly, functional connectivity strengths between seed regions of interests for different isoflurane levels showed comparable trends to that of power analyses (Figure 4). The universal effects of different isoflurane levels on functional connectivity between functionally highly correlated hand–hand pairs and uncorrelated hand–face ROI pairs further support the conclusion that the suppressive effects of isoflurane are not functionally selective. Our findings here highlight that isoflurane causes nonfunctionally selective and more general suppressive effects on local fine‐scale cortical circuits.

### 4.2 Anesthesia and its effects on brain networks

Anesthesia is widely used in resting‐state fMRI studies in animals (both rodents and non‐human primates) as it provides investigators with numerous advantages. Firstly, it establishes a more controlled system with reduced motion‐related effects and physiological stress, and it eliminates trainings required for awake animals. More importantly, anesthesia also allows for the modulation of neural activity and exploitation of neurovascular decoupling to understand deeper into the biophysical basis underlying spontaneous fluctuations and functional connectivity (see a review, Hutchison & Everling, 2012). That being said, properties and effects of anesthesia on resting‐state functional connectivity remain unclear and may even introduce confounding effects (discussed further later).

While the effects of anesthesia are yet to be fully understood, possible mechanisms have been proposed on how anesthesia affects the brain network (Alkire et al., 2008). One is the loss or reduction in integration as anesthesia breaks down synchronization between different cortical regions of the brain, resulting in decreased correlational patterns (Deshpande, Kerssens, Sebel, & Hu, 2010; Hutchison et al., 2014; Lu et al., 2007). On the other hand, studies have also demonstrated the loss of specificity in cortical regions showing synchronized rsfMRI signal fluctuations with increased anesthesia levels. In other words, the brain's capability for encoding information is disrupted with a loss of segregation (Liu, Zhu, Zhang, & Chen, 2011; Liu, Zhu, et al., 2013). Our present findings support the first mechanism. Under high isoflurane level (1.25%), BOLD signals resulted in lower spontaneous signal fluctuations as well as weaker inter‐regional in functionally related but distinct S1 subregions. Our findings are also in line with the other investigation on graded effects of isoflurane on non‐human primates. Hutchison et. al made use of methods such as independent component analysis (ICA) and dynamic state detections to observe decrease in functional connectivity patterns in the brain's overall functional architecture (Hutchison et al., 2014). Extending and building upon these observations, we showed that isoflurane also causes nonfunctionally selective and more general suppressive effects on specific local fine‐scale cortical circuits (digit‐to‐digit regions within S1 cortex).

It has also been shown in humans that light levels of isoflurane induce unconsciousness that is reflected in electroencephalography recordings with transition into slower wave oscillations. At deeper anesthesia levels, burst‐suppression patterns begin to arise before electrical silence at even higher levels (Hutchison et al., 2014; Sloan, 1998; Swank & Watson, 1949). Specifically, in macaques, an anesthesia level of 0.90–1.00% has been shown to produce continuous low‐frequency oscillations while at deeper anesthesia levels between 1.25% and 1.50%, this activity shifts to burst suppression (Vincent et al., 2007). Moreover, our recent study in squirrel monkeys shows a strong relationship between correlations in rsfMRI fluctuations and low/middle frequency range of local field potentials under the isoflurane range of 0.6–1.1% (Wilson et al., 2016). Indeed, this study provides novel and additional information about the extent of isoflurane on brain rsfMRI signals.

### 4.3 Isoflurane and possible confounds

Isoflurane is considered the most widely used anesthetic in non‐human primate studies; thanks to its robustness and suitability for survival studies. Specifically, isoflurane has been reported to interfere with neural pathways through the reduction of presynaptic excitability by blockading sodium channels (Hemmings, 2009). Others have also observed the potentiation of GABA_A_ as well as NMDA, nicotinic and acetylcholine receptor inhibitions (Dickinson et al., 2007; Hentschke, Schwarz, & Antkowiak, 2005; Violet, Downie, Nakisa, Lieb, & Franks, 1997; for reviews see Franks, 2008 and Masamoto & Kanno, 2012). However, how reductions of neuronal electrical activity (at the synaptic level) influence rsfMRI signal fluctuations remains unknown.

Our choice of the three light‐to‐moderate levels of isoflurane in this study is based on three main factors. Firstly, our previous fMRI and optical imaging studies of somatosensory system in monkeys have demonstrated robust and detectable functional imaging signals that are sensitive to subtle and natural tactile stimuli in the primary (area 3b) and secondary (S2) somatosensory cortices as well as in high‐order anterior cingulate cortex under ~1.0% isoflurane. Moreover, fMRI signal fluctuations detected were also able to capture changes in afferent inputs caused by spinal cord injury (Chen, Qi, & Kaas, 2012; Yang et al., 2014). Secondly, under such range of isoflurane, neurons also responded robustly in a functionally selective manner to peripheral stimuli (Chen et al., 2012; Qi, Chen, & Kaas, 2011). Thirdly, the meso‐scale resting‐state fMRI networks within the somatosensory cortices that we proposed in this study were also detectable between 0.5% to 1.0% isoflurane. These findings led us to believe that under light and moderate levels of isoflurane anesthesia, neuronal activities and functional imaging signals are well retained, and therefore provides an excellent model for studying the neuronal basis of fMRI signals. Taken together, the three levels of isoflurane were selected within the range because functionally relevant neuronal signals in both somatosensory and high cognitive cortices are preserved.

Neurovascular decoupling also arises with higher dosages of isoflurane. With elevated levels of isoflurane, metabolic rates as well as CBF and oxygenation can decrease due to hypotensive effects. As a result, BOLD contrast‐to‐noise ratio may decrease, and thus artificially lower spontaneous fluctuations and correlation values (Hutchison et al., 2014). Under our experimental conditions with light‐to‐moderate isoflurane anesthesia, we have recently found that differences in inter‐regional rsfMRI FC within S1 digit regions correlate with the differences in inter‐regional coherence level of local field potentials (Wilson et al., 2016). This finding indicates that rsFC remains tightly associated with neuronal electrical activity at the anesthesia levels that were examined in this study. Several reports have also provided evidences supporting a close relationship between rsfMRI functional connectivity and neuronal activity (Liu et al., 2011; Lu et al., 2007; Shmuel & Leopold, 2008). Notably, despite physiological signals being continuously monitored, the sampling rate of the recordings was insufficient for accurate regression from the time series. Although the trends observed are deemed unlikely to be driven by such effects (discussed further below), physiological signals (i.e., heartbeat, respiration, and CO2) can be used as covariates in statistical analysis in future studies to minimize confounding effects. In addition, the presence of nitrous oxide (also an anesthetic) may complicate or confound our observations. However, since the level of nitrous oxide was kept constant, it is very unlikely that the observed isoflurane effects are driven by the presence of nitrous oxide. Nevertheless, the potential contributions of interactions between nitrous oxide and isoflurane need to be further explored. To fully understand the complex interaction between neural activity and hemodynamic process under different anesthesia levels, combinations of electrical recordings and fMRI studies will be essential. Indeed, existing evidence from several studies with EEG and fMRI recordings in both anesthetized rodents and non‐human primates support close correlations between electrical activity and functional connectivity (Liu, Pillay, et al., 2013; Lu et al., 2007; Vincent et al., 2007).

Even though simultaneous assessment of functional connectivity and electrical activity was not performed in our monkey experiments, several observations in our study have indicated the trends observed were not solely due to the vascular effect of isoflurane. The correlation r values between area 1 and 3b (Figure 5) at 0.5% level reached up to a maximum of 0.92, indicating high BOLD signal variation compared to noise. Furthermore, the mean inter‐regional correlations between the ROIs and face control region did not vary across different isoflurane levels, and the patterns of inter‐regional correlation differences were preserved across different levels. Our careful selection of the muscle control region also demonstrated a relatively constant trend of fALFF/ALFF, with no statistical differences throughout different anesthesia levels. Vascular effects are also expected to be present in the muscle regions, so our findings suggest that the BOLD signal changes we observed are most likely driven by hemodynamic effects corresponding to changes in neural activity. Although other studies and our results have pointed toward the unlikeliness of vascular confounds being a major contributor, findings presented here still need to be interpreted with caution.

Another possible confound is the motion of the animals across different isoflurane levels. However, this appears unlikely as motion was minimized in this preparation, and motion parameters and muscle signals were regressed out before performing inter‐regional correlation analyses. This further reduces the possibility of artificially altering correlation values due to physiological or global SNR changes unrelated to neural activity.

### 4.4 Appropriate isoflurane level range

The decreasing trend in functional connectivity highlights the importance of selecting an appropriate isoflurane level. Specifically, a relatively large dip in correlation value is observed between 0.875% and 0.5% isoflurane levels. This suggests that an appropriate isoflurane level for imaging of the SI region should be maintained as stable as possible during functional scans. Indeed, our previous rsFC measures were robust and sensitive in differentiating functionally distinct fine‐scale sensory circuits in S1 cortices within such isoflurane range (Chen et al., 2007, 2012; Yang et al., 2014). While several reports have also demonstrated breakdown of circuits at specific anesthesia levels (Hutchison et al., 2014; Peltier et al., 2005; Vincent et al., 2007), consensus on an appropriate anesthesia range for functional connectivity studies is yet to be established. In fact, forming such standard across studies would be difficult as it is dependent on numerous factors such as the type of anesthetics and species of interest.

## 5 Conclusion

Changing the anesthesia level altered the power measures and apparent functional connectivity of resting‐state fMRI signals in specific regions of the S1 cortex. Given the known suppressive effects of anesthesia on neuronal activity, our observation of decreased functional connectivity to increased isoflurane levels supports the neuronal basis of rsfMRI signals. This paper also provides a quantitative evaluation of functional connectivity between functionally related digits regions within the S1 region across different anesthesia levels. The evaluation of an appropriate isoflurane level will be helpful to avoid affecting the intrinsic functional relations between various regions in a resting state.

## Conflict of Interests

None declared.

## References

[brb3591-bib-0001] Alkire, M. T. , Alkire, M. T. , Hudetz, A. G. , Hudetz, A. G. , Tononi, G. , & Tononi, G. (2008). Consciousness and anesthesia. Science, 322, 876–880. doi:10.1126/science.1149213 18988836 10.1126/science.1149213 PMC2743249

[brb3591-bib-0002] Barttfeld, P. , Uhrig, L. , Sitt, J. D. , Sigman, M. , Jarraya, B. , & Dehaene, S. (2014). Signature of consciousness in the dynamics of resting‐state brain activity. Proceedings of the National Academy of Sciences of the United States of America, 112, 201418031. doi:10.1073/pnas.1418031112 10.1073/pnas.1418031112 PMC4311826 25561541

[brb3591-bib-0003] Biswal, B. , Yetkin, F. Z. , Haughton, V. M. , & Hyde, J. S. (1995). Functional connectivity in the motor cortex of resting human brain using echo‐planar MRI. Magnetic Resonance in Medicine, 34, 537–541. doi:10.1002/mrm.1910340409 8524021 10.1002/mrm.1910340409

[brb3591-bib-0004] Chen, L. , Mishra, A. , Newton, A. T. , Morgan, V. L. , Stringer, E. A. , Rogers, B. P. , & Gore, J. C. (2011). Fine‐scale functional connectivity in somatosensory cortex revealed by high‐resolution fMRI. Magnetic Resonance in Medicine, 29, 1330–1337. doi:10.1016/j.mri.2011.08.001 10.1016/j.mri.2011.08.001 PMC3285506 21982165

[brb3591-bib-0005] Chen, L. M. , Mishra, A. , Yang, P.‐F. , Wang, F. , & Gore, J. C. (2015). Injury alters intrinsic functional connectivity within the primate spinal cord. Proceedings of the National Academy of Sciences of the United States of America, 112, 5991–5996. PMID: 25902510. doi:10.1073/pnas.1424106112 25902510 10.1073/pnas.1424106112 PMC4434735

[brb3591-bib-0006] Chen, L. M. , Qi, H.‐X. , & Kaas, J. H. (2012). Dynamic reorganization of digit representations in somatosensory cortex of nonhuman primates after spinal cord injury. Journal of Neuroscience, 32, 14649–14663. doi:10.1523/JNEUROSCI.1841‐12.2012 23077051 10.1523/JNEUROSCI.1841-12.2012 PMC3498942

[brb3591-bib-0007] Chen, L. M. , Turner, G. H. , Friedman, R. M. , Zhang, N. , Gore, J. C. , Roe, A. W. , & Avison, M. J. (2007). High‐resolution maps of real and illusory tactile activation in primary somatosensory cortex in individual monkeys with functional magnetic resonance imaging and optical imaging. Journal of Neuroscience, 27, 9181–9191. doi:10.1523/JNEUROSCI.1588‐07.2007 17715354 10.1523/JNEUROSCI.1588-07.2007 PMC6672200

[brb3591-bib-0008] Deshpande, G. , Kerssens, C. , Sebel, P. S. , & Hu, X. (2010). Altered local coherence in the default mode network due to sevoflurane anesthesia. Brain Research, 1318, 110–121. doi:10.1016/j.brainres.2009.12.075 20059988 10.1016/j.brainres.2009.12.075 PMC2845285

[brb3591-bib-0009] Dickinson, R. , Peterson, B. K. , Banks, P. , Simillis, C. , Martin, J. C. S. , Valenzuela, C. A. , … Franks, N. P. (2007). Competitive inhibition at the glycine site of the N‐methyl‐D‐aspartate receptor by the anesthetics xenon and isoflurane: Evidence from molecular modeling and electrophysiology. Anesthesiology, 107, 756–767. doi:10.1097/01.anes.0000287061.77674.71 18073551 10.1097/01.anes.0000287061.77674.71

[brb3591-bib-0010] Fox, M. D. , Corbetta, M. , Snyder, A. Z. , Vincent, J. L. , & Raichle, M. E. (2006). Spontaneous neuronal activity distinguishes human dorsal and ventral attention systems. Proceedings of the National Academy of Sciences of the United States of America, 103, 10046–10051. doi:10.1073/pnas.0604187103 16788060 10.1073/pnas.0604187103 PMC1480402

[brb3591-bib-0011] Fox, M. D. , & Greicius, M. (2010). Clinical applications of resting state functional connectivity. Frontiers in Systems Neuroscience, 4, 19. doi:10.3389/fnsys.2010.00019 20592951 10.3389/fnsys.2010.00019 PMC2893721

[brb3591-bib-0012] Franks, N. P. (2008). General anaesthesia: From molecular targets to neuronal pathways of sleep and arousal. Nature Reviews Neuroscience, 9, 370–386. doi:10.1038/nrn2372 18425091 10.1038/nrn2372

[brb3591-bib-0013] Grandjean, J. , Schroeter, A. , Batata, I. , & Rudin, M. (2014). Optimization of anesthesia protocol for resting‐state fMRI in mice based on differential effects of anesthetics on functional connectivity patterns. NeuroImage, 102, 838–847. doi:10.1016/j.neuroimage.2014.08.043 25175535 10.1016/j.neuroimage.2014.08.043

[brb3591-bib-0014] Greicius, M. D. , Kiviniemi, V. , Tervonen, O. , Vainionpää, V. , Alahuhta, S. , Reiss, A. L. , & Menon, V. (2008). Persistent default‐mode network connectivity during light sedation. Human Brain Mapping, 29, 839–847. doi:10.1002/hbm.20537 18219620 10.1002/hbm.20537 PMC2580760

[brb3591-bib-0015] Heinke, W. , & Koelsch, S. (2005). The effects of anesthetics on brain activity and cognitive function. Current Opinion in Anaesthesiology, 18, 625–631. doi:10.1097/01.aco.0000189879.67092.12 16534303 10.1097/01.aco.0000189879.67092.12

[brb3591-bib-0016] Hemmings, H. C. (2009). Sodium channels and the synaptic mechanisms of inhaled anaesthetics. British Journal of Anaesthesia, 103, 61–69. doi:10.1093/bja/aep144 19508978 10.1093/bja/aep144 PMC2700013

[brb3591-bib-0017] Hentschke, H. , Schwarz, C. , & Antkowiak, B. (2005). Neocortex is the major target of sedative concentrations of volatile anaesthetics: Strong depression of firing rates and increase of GABAA receptor‐mediated inhibition. European Journal of Neuroscience, 21, 93–102. doi:10.1111/j.1460‐9568.2004.03843.x 15654846 10.1111/j.1460-9568.2004.03843.x

[brb3591-bib-0018] Hutchison, R. M. , & Everling, S. (2012). Monkey in the middle: Why non‐human primates are needed to bridge the gap in resting‐state investigations. Frontiers in Neuroanatomy, 6, 29. doi:10.3389/fnana.2012.00029 22855672 10.3389/fnana.2012.00029 PMC3405297

[brb3591-bib-0019] Hutchison, R. M. , Hutchison, M. , Manning, K. Y. , Menon, R. S. , & Everling, S. (2014). Isoflurane induces dose‐dependent alterations in the cortical connectivity profiles and dynamic properties of the brain's functional architecture. Human Brain Mapping, 35, 5754–5775. doi:10.1002/hbm.22583 25044934 10.1002/hbm.22583 PMC6869297

[brb3591-bib-0020] Jonckers, E. , Palacios, R. D. , Shah, D. , Guglielmetti, C. , Verhoye, M. , & Van Der Linden, A. (2014). Different anesthesia regimes modulate the functional connectivity outcome in mice. Magnetic Resonance in Medicine, 72, 1103–1112. doi:10.1002/mrm.24990 24285608 10.1002/mrm.24990

[brb3591-bib-0021] Lee, M. H. , Smyser, C. D. , & Shimony, J. S. (2013). Resting‐state fMRI: A review of methods and clinical applications. AJNR. American Journal of Neuroradiology, 34, 1866–1872. doi:10.3174/ajnr.A3263 22936095 10.3174/ajnr.A3263 PMC4035703

[brb3591-bib-0022] Liu, J. V. , Hirano, Y. , Nascimento, G. C. , Stefanovic, B. , Leopold, D. A. , & Silva, A. C. (2013). FMRI in the awake marmoset: Somatosensory‐evoked responses, functional connectivity, and comparison with propofol anesthesia. NeuroImage, 78, 186–195. doi:10.1016/j.neuroimage.2013.03.038 23571417 10.1016/j.neuroimage.2013.03.038 PMC3778909

[brb3591-bib-0023] Liu, X. , Pillay, S. , Li, R. , Vizuete, J. A. , Pechman, K. R. , Schmainda, K. M. , & Hudetz, A. G. (2013). Multiphasic modification of intrinsic functional connectivity of the rat brain during increasing levels of propofol. NeuroImage, 83, 581–592. doi:10.1016/j.neuroimage.2013.07.003 23851326 10.1016/j.neuroimage.2013.07.003 PMC3815996

[brb3591-bib-0024] Liu, X. , Zhu, X. H. , Zhang, Y. , & Chen, W. (2011). Neural origin of spontaneous hemodynamic fluctuations in rats under burst‐suppression anesthesia condition. Cerebral Cortex, 21, 374–384. doi:10.1093/cercor/bhq105 20530220 10.1093/cercor/bhq105 PMC3020581

[brb3591-bib-0025] Liu, X. , Zhu, X. H. , Zhang, Y. , & Chen, W. (2013). The change of functional connectivity specificity in rats under various anesthesia levels and its neural origin. Brain Topography, 26, 363–377. doi:10.1007/s10548‐012‐0267‐5 23208517 10.1007/s10548-012-0267-5 PMC3622140

[brb3591-bib-0026] Lu, H. , Zuo, Y. , Gu, H. , Waltz, J. A. , Zhan, W. , Scholl, C. A. , … Stein, E. A. (2007). Synchronized delta oscillations correlate with the resting‐state functional MRI signal. Proceedings of the National Academy of Sciences of the United States of America, 104, 18265–18269. doi:10.1073/pnas.0705791104 17991778 10.1073/pnas.0705791104 PMC2084331

[brb3591-bib-0027] Lv, P. , Xiao, Y. , Liu, B. , Wang, Y. , Zhang, X. , Sun, H. , … Lui, S. (2016). Dose‐dependent effects of isoflurane on regional activity and neural network function : A resting‐state fMRI study of 14 rhesus monkeys An observational study. Neuroscience Letters, 611, 116–122. 26633103 10.1016/j.neulet.2015.11.037

[brb3591-bib-0028] Masamoto, K. , & Kanno, I. (2012). Anesthesia and the quantitative evaluation of neurovascular coupling. Journal of Cerebral Blood Flow and Metabolism, 32, 1233–1247. doi:10.1038/jcbfm.2012.50 22510601 10.1038/jcbfm.2012.50 PMC3390804

[brb3591-bib-0029] Peltier, S. J. , Kerssens, C. , Hamann, S. B. , Sebel, P. S. , Byas‐Smith, M. , & Hu, X. (2005). Functional connectivity changes with concentration of sevoflurane anesthesia. NeuroReport, 16, 285–288. doi:10.1097/00001756‐200502280‐00017 15706237 10.1097/00001756-200502280-00017

[brb3591-bib-0030] Qi, H.‐X. , Chen, L. M. , & Kaas, J. H. (2011). Reorganization of somatosensory cortical areas 3b and 1 after unilateral section of dorsal columns of the spinal cord in squirrel monkeys. Journal of Neuroscience, 31, 13662–13675. doi:10.1523/JNEUROSCI.2366‐11.2011 21940457 10.1523/JNEUROSCI.2366-11.2011 PMC3183096

[brb3591-bib-0031] Shmuel, A. , & Leopold, D. A. (2008). Neuronal correlates of spontaneous fluctuations in fMRI signals in monkey visual cortex: implications for functional connectivity at rest. Human Brain Mapping, 761, 751–761. doi:10.1002/hbm.20580 10.1002/hbm.20580 PMC6870786 18465799

[brb3591-bib-0032] Sloan, T. B. (1998). Anesthetic effects on electrophysiologic recordings. Journal of Clinical Neurophysiology, 15, 217–226. doi:10.1097/00004691‐199805000‐00005 9681559 10.1097/00004691-199805000-00005

[brb3591-bib-0033] Spitzer, M. , Wildenhain, J. , Rappsilber, J. , & Tyers, M. (2014). BoxPlotR: A web tool for generation of box plots. Nature Methods, 11, 121–122. 24481215 10.1038/nmeth.2811 PMC3930876

[brb3591-bib-0034] Swank, R. , & Watson, C. (1949). Effects of barbiturates and ether on spontaneous electrical activity of dog brain. Journal of Neurophysiology, 12, 137–160. 18114367 10.1152/jn.1949.12.2.137

[brb3591-bib-0035] Vincent, J. L. , Patel, G. H. , Fox, M. D. , Snyder, A. Z. , Baker, J. T. , Van Essen, D. C. , … Raichle, M. E. (2007). Intrinsic functional architecture in the anaesthetized monkey brain. Nature, 447, 83–86. doi:10.1038/nature05758 17476267 10.1038/nature05758

[brb3591-bib-0036] Violet, J. M. , Downie, D. L. , Nakisa, R. C. , Lieb, W. R. , & Franks, N. P. (1997). Differential sensitivities of mammalian neuronal and muscle nicotinic acetylcholine receptors to general anesthetics. Anesthesiology, 86, 866–874. doi:10.1097/00000542‐199704000‐00017 9105231 10.1097/00000542-199704000-00017

[brb3591-bib-0037] Wang, Z. , Chen, L. M. , Négyessy, L. , Friedman, R. M. , Mishra, A. , Gore, J. C. , & Roe, A. W. (2013). The relationship of anatomical and functional connectivity to resting‐state connectivity in primate somatosensory cortex. Neuron, 78, 1116–1126. doi:10.1016/j.neuron.2013.04.023 23791200 10.1016/j.neuron.2013.04.023 PMC3723346

[brb3591-bib-0038] Wilson, G. H. , Yang, P.‐F. , Gore, J. C. , & Chen, L. M. (2016). Correlated inter‐regional variations in low frequency local field potentials and resting state BOLD signals within S1 cortex of monkeys. Human Brain Mapping, 37, 2755–2766. doi: 10.1002/hbm.23207 27091582 10.1002/hbm.23207 PMC4945372

[brb3591-bib-0039] Yang, P.‐F. , Qi, H.‐X. , Kaas, J. H. , & Chen, L. M. (2014). Parallel functional reorganizations of somatosensory areas 3b and 1, and S2 following spinal cord injury in squirrel monkeys. Journal of Neuroscience, 34, 9351–9363. doi:10.1523/JNEUROSCI.0537‐14.2014 25009268 10.1523/JNEUROSCI.0537-14.2014 PMC4087212

[brb3591-bib-0040] Zang, Y.‐F. , He, Y. , Zhu, C.‐Z. , Cao, Q.‐J. , Sui, M.‐Q. , Liang, M. , … Wang, Y.‐F. (2007). Altered baseline brain activity in children with ADHD revealed by resting‐state functional MRI. Brain and Development, 29, 83–91. doi:10.1016/j.braindev.2006.07.002 16919409 10.1016/j.braindev.2006.07.002

[brb3591-bib-0041] Zou, Q. , Wu, C. W. , Stein, E. A. , Zang, Y. , & Yang, Y. (2009). Static and dynamic characteristics of cerebral blood flow during the resting state. NeuroImage, 48, 515–524. doi:10.1016/j.neuroimage.2009.07.006 19607928 10.1016/j.neuroimage.2009.07.006 PMC2739419

[brb3591-bib-0042] Zuo, X. N. , Di Martino, A. , Kelly, C. , Shehzad, Z. E. , Gee, D. G. , Klein, D. F. , … Milham, M. P. (2010). The oscillating brain: Complex and reliable. NeuroImage, 49, 1432–1445. doi:10.1016/j.neuroimage.2009.09.037 19782143 10.1016/j.neuroimage.2009.09.037 PMC2856476

